# Distanciamento social por Covid 19: repercussão na rotina de universitários**

**DOI:** 10.15649/cuidarte.2093

**Published:** 2022-08-20

**Authors:** Luciano Fiorentin, Vilma Beltrame

**Affiliations:** 1 Universidade do Oeste de Santa Catarina UNOESC. Joaçaba, Santa Catarina. Brasil. Email: fiorentinl@yahoo.com.br. Universidade do Oeste de Santa Catarina Universidade do Oeste de Santa Catarina Joaçaba Santa Catarina Brazil fiorentinl@yahoo.com.br; 2 Universidade do Oeste de Santa Catarina UNOESC. Joaçaba, Santa Catarina. Brasil. Email: vilma.beltrame@unoesc.edu.br Universidade do Oeste de Santa Catarina Universidade do Oeste de Santa Catarina Joaçaba Santa Catarina Brazil vilma.beltrame@unoesc.edu.br

**Keywords:** Infecções por Coronavirus, Pandemia, Saúde do Estudante, Isolamento Social, Coronavirus Infections, Pandemic, Student Health, Social Isolation, Infecciones por Coronavirus, Pandemia, Salud del Estudiante, Aislamiento social

## Abstract

**Introdução:**

Necessitando de medidas de combate à pandemia por SARSCoV-2, a Organização Mundial da Saúde orientou o distanciamento social como estratégia para mitigar seus impactos. Dentre as medidas adotadas para evitar aglomerações, universidades se adaptaram para modalidade de ensino online. O objetivo é refletir sobre a influência do distanciamento social por covid-19, na rotina de estudantes de ciências da saúde.

**Materiais e Métodos:**

É uma revisão integrativa, com buscas nas bases de dados Pubmed, MEDLINE, LILACS, WHO COVID, bioRxiv, e medRxiv, nos sites da Pubmed, BVS e Portal de buscas da Organização Mundial da Saúde para covid-19, com descritores nas seguintes combinações: “Infecções por coronavírus e estudantes de ciências de saúde” e “Covid-19 e estudantes de ciências de saúde”. Inicialmente encontrou-se 1069 artigos.

**Resultados:**

Após refinamento 23 artigos compuseram o estudo. Os resultados foram categorizados como 1-desordens nas competências emocionais, 2-ensino, 3-alimentação, sono e atividade física e 4-efeitos de natureza diversas. Manifestações frequentes: ansiedade, depressão, estresse, incerteza e angústia, mudanças no sono e atividade física. Vantagem ou desvantagem do ensino online, teve percentual de 50% cada.

**Conclusão:**

Conhecer como o distanciamento social influenciou na rotina dos estudantes é relevante para que as universidades desenvolvam programas de suporte para as necessidades apresentadas na realidade atual, e consigam projetar programas de enfrentamento para futuras crises epidêmicas, auxiliando na redução dos impactos resultantes.

## Abstract (en)

**Introduction:**

In response to the SARS-CoV-2 pandemic, the World Health Organization recommended social distancing as a strategy to mitigate the impacts of the coronavirus disease. Among measures adopted to avoid crowds, universities then switched to virtual education. The objective is to reflect on the influence of social distancing during COVID-19 on the daily routine of health sciences students.

**Materials and methods:**

A systematic literature review was conducted by searching on PubMed, MEDLINE, LILACS, bioRxiv, medRxiv, as well as BVS and the World Health Organization site for COVID-19 (WHO COVID-19) databases, using combined descriptors such as “coronavirus infections and health science students” and “COVID-19 and health science students”. A total of 1069 articles were initially found, being later refined to 23 for the study.

**Results:**

were classified into 1) emotional competence disorder, 2) education, 3) eating, sleep and physical activity, and 4) various kind of impacts with frequent manifestations of anxiety, depression, stress, uncertainty distress and changes in physical activity and sleep habits. Student dropout in virtual programs is about 50% in all cases.

**Conclusions:**

Knowing how social distancing influenced the daily routine of college students is important for universities to develop support programs tailored to current reality needs and implement future epidemic preparedness programs, helping reduce possible impacts.

## Resumen

**Introducción:**

Ante la necesidad de combatir la pandemia por SARS-CoV-2, la Organización Mundial de la Salud recomendó el distanciamiento social como estrategia para mitigar los impactos de la enfermedad. Entre las medidas adoptadas para evitar aglomeraciones, las universidades se han adaptado a la modalidad de educación virtual. El objetivo es reflexionar sobre la influencia del distanciamiento social por COVID-19 en la rutina de los estudiantes de ciencias de la salud.

**Materiales y métodos:**

Se realizó una revisión sistemática de la literatura a través de búsquedas en PubMed, MEDLINE, LILACS, bioRxiv, medRxiv, así como en BVS y el portal de búsqueda de la Organización Mundial de la Salud para COVID-19 (WHO COVID-19), con descriptores combinados de la siguiente forma: “infecciones por coronavirus y estudiantes de ciencias de la salud” y “COVID-19 y estudiantes de ciencias de la salud”. Se encontraron 1069 artículos inicialmente, que luego fueron depurados a 23 para conformar el estudio.

**Resultados:**

Los resultados se categorizaron en: 1) trastorno en las competencias emocionales, 2) educación, 3) alimentación, sueño y actividad física, y 4) efectos de diversa índole, con manifestaciones frecuentes de ansiedad, depresión, estrés, incertidumbre y angustia, y cambios en el sueño y la actividad física. La deserción escolar en entornos virtuales es del 50% en todos los casos.

**Conclusiones:**

Es importante saber la forma en la que el distanciamiento social influyó en la rutina de los estudiantes, para que así las universidades logren desarrollar programas de apoyo que se ajusten a las necesidades de la realidad actual e implementar programas de respuesta a futuras crisis epidemiológicas, lo que ayudaría a reducir los posibles impactos.

## Introdução

Após a identificação e registro do primeiro caso de infecção da Síndrome Respiratória Aguda Grave por Coronavirus - SARS-CoV-2 causador da doença Covid-19 em dezembro de 2019^1^, o mundo vive uma pandemia, a qual em suas marcas, já acumula centenas de milhares de infecções e mortes pelo mundo.^2^

A rápida disseminação, dada pelo elevado poder de transmissibilidade, e sem mecanismos de combate, o vírus se espalhou livremente pelo mundo obrigando autoridades governamentais e sanitárias a adotarem medidas epidemiológicas restritivas, como o distanciamento social, com o propósito de mitigar a epidemia.^3^^,^^4^

Dentre as medidas de distanciamento social, foi orientado pela OMS a necessidade de restrição de contato entre humanos, evitar eventos que proporcionassem aglomeração de pessoas, fechamento temporário de fronteiras, suspensão de trabalho e ou atividades não essenciais, incluindo escolas e universidades.^5^

As medidas de distanciamento social foram estratégias epidemiológicas, utilizadas para combater a Covid-19^5^, no entanto, essas medidas restringem os contatos socialmente constituídos. Por mais que a causa seja justificável, os efeitos psicológicos tendem a se apresentar com respostas negativas, como estresse, medo e ansiedade.^6^

Para estudantes de ciências da saúde, em que vivenciaram as universidades fechadas e migrando para novas alternativas de ensino, como a modalidade online, os reflexos que se apresentam é de uma realidade de dúvidas relacionadas à bruscas transformações sobre o ensino e incertezas sobre o futuro, resultante dessa crise pandêmica.^7^

Os alunos de ciências da saúde possuem como requisito em sua formação, períodos de aulas essencialmente presenciais, momentos de práticas e estágios, que foram interrompidos pelo distanciamento social. Também a suspensão das atividades não essenciais, que era o local de trabalho de alguns estudantes, que viram sua fonte de renda ameaçada ou suspensa, passaram a viver a insegurança financeira. Diante disso levanta-se o questionamento de como o distanciamento social por Covid-19 influenciou a rotina acadêmica de estudantes de ciências da saúde?

Acentua-se essa busca por respostas outro fator considerado relevante, ou seja, o fato deles serem os futuros profissionais da saúde, e em situações de crise de saúde pública, a exemplo da pandemia por Covid-19, estão ou estarão na linha de frente, com elevado grau de exposição para o adoecimento. E dessa forma, esse estudo possui como objetivo, refletir sobre a influência do distanciamento social por Covid-19, na rotina de estudantes de ciências da saúde.

## Materiais e métodos

Este estudo é uma revisão integrativa da literatura, resultante de um recorte de dissertação, onde buscou-se artigos que descrevessem sobre o distanciamento social por Covid-19, e as influências que suas restrições tiveram na rotina acadêmica de estudantes de ciências da saúde.

As etapas formam sequencialmente seguidas conforme sugerem Souza; Silva; Carvalho^8^: Elaboração da pergunta norteadora; Busca ou amostragem na literatura; Coleta de dados; Análise crítica dos estudos incluídos; Discussão dos resultados e Apresentação da revisão integrativa.

Utilizou-se como estratégia de busca as combinações de descritores “Infecções por coronavirus e estudantes de ciências de saúde”; “Coronavirus infections and health science students”; “Covid-19 e estudantes de ciências de saúde” e “covid-19 and health science students”, a partir das bases de dados Pubmed, MEDLINE, LILACS, WHO COVID, bioRxiv, e medRxiv, através dos portais de buscas da Pubmed, Biblioteca Virtual em Saúde (BVS) e Portal de buscas da OMS para covid-19. A busca foi realizada nos dias 03 e 04 de janeiro de 2021. Os filtros usados foram ‘textos completos” e “últimos 5 anos”

Após a busca, foi exportado arquivo total em formato “RIS” para artigos encontrados na BVS e Portal de buscas da OMS para covid-19, e “medline” para os artigos encontrados na plataforma de busca da Pubmed. Foi utilizado auxílio do StArt (gerenciador de revisão sistemática de bibliografia) versão (v. 3.3 beta 03), para seleção e extração dos artigos. Após os artigos extraídos, foi utilizado o software Zotero (v. 5.0.89) como gerenciador de referências. Após importação para sistema foram encontrados o total de 1069 publicações, destes 133 o sistema Start identificou como duplicados, ficando 936 para fase de seleção.^9^

Os arquivos extraídos que compõe o banco de dados da pesquisa, estão armazenados em repositório público, mas de acesso restrito aos pesquisadores.

**Figura 01:**
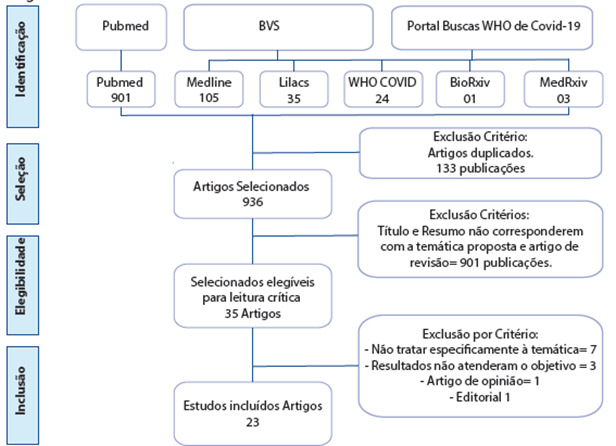
Fluxo de informações da revisão integrativa

Conforme apresentado na figura 01, as informações seguiram o mesmo fluxo PRISMA, recomendado por Liberati et. al,^10^ a ser seguido em revisões sistemáticas de literatura. Com esse fluxo é possível apresentar com transparência do caminho percorrido até a definição final dos artigos incluídos.

Fonte: Autores

Para a extração dos dados, utilizou-se um fichamento das informações encontradas nos artigos selecionados para o estudo (Tabela 1), e que, a partir das influências que os estudos mostraram sobre a rotina dos estudantes em virtude do distanciamento social devido a Covid-19, estruturou-se categorias com informações quantificáveis, melhorando a visibilidade dos desfechos encontrados. A análise e interpretação ocorreu à luz da temática proposta, e apresentada de forma descritiva

## Resultados

Para apresentação das características dos estudos selecionados, e que compõem esta revisão integrativa, foi elaborado a Tabela 1, a qual traz informações sobre o ano de publicação e o periódico, a autoria do artigo, título, delineamento e amostra, objetivo e conclusão.

De acordo com a tabela 01, os 23 artigos selecionados foram publicados no ano de 2020. Esse fato ocorre devido ao período de surgimento do surto pelo novo coronavirus, ter sido registrado em Wuhan, China, em dezembro de 2019,^11^ e que após reconhecimento pela OMS como pandemia^12^, a mesma está em andamento com abrangência mundial.

**Tabela 1:** Características dos estudos selecionados, relativas ao ano, periódico, autoria, título, delineamento e amostra, objetivo e conclusão.

Nº	Ano publicado e periódico	Autor	Título	Delineamento e amostra	Objetivo	Conclusão
A1	2020 European Journal of Dentistry	Bashir et al.^13^	A análise do impacto psicológico da nova pandemia de COVID-19 em estudantes de ciências da saúde: uma pesquisa global	Estudo transversal Amostra: 523 estudantes de ciências da saúde	Avaliar a ansiedade e a depressão em meio à nova pandemia da doença por coronavírus 19 (COVID-19) em estudantes de ciências da saúde em todo o mundo.	Quarentena devido a Covid-19 trouxe impactos negativos para saúde mental de Estudantes de ciências da Saúde
A2	2020 BMC Medical Education	Al-Balas et al.^14^	Ensino à distância em educação médica clínica em meio à pandemia de COVID-19 na Jordânia: situação atual, desafios e perspectivas	Estudo transversal Amostra: 652 Estudantes 4ºao 6º de medicina	Explorar a situação do E-learning a distância entre estudantes de medicina durante seus anos clínicos e identificar possíveis desafios, limitações, satisfação e perspectivas para essa abordagem de aprendizagem.	Os estudantes apontaram benefícios ao ensino a distância, mas preferem o ensino misto. Identi_cado grandes desa_os para desenvolver habilidades médicas no ensino a distância.
A3	2020 Nurse Education Today	Collado-Boira et al.^15^	Estudo transversal Amostra: 652 Estudantes 4ºao 6º de medicina	Abordagem qualitativa fenomenológica Amostra: 62 alunos do último ano de enfermagem e medicina da Universidade Jaime I (Espanha)	Estudar, numa abordagem qualitati-va fenomenológica, as percepções dos alunos nesta situação real excepcional	Os alunos estavam dispostos a ajudar na situação de crise A experiência foi demonstrada por impressões negativas em relação a situação em que foram apresentados, em especial risco iminente de morte
A4	2020 Radiography	Courtier et al.^16^	Expectativas dos estudantes de radiografia terapêutica no País de Gales sobre a transição para a prática durante a pandemia de Covid-19 como inscritos no registro temporário do HCPC	Estudo qualitativo 11 alunos do último ano de radiologia terapêutica	Explorar os sentimentos e expectativas dos alunos sobre o início do trabalho durante a pandemia, em uma coorte que havia sido removida de seus estágios educacionais em departamentos do NHS	A Pandemia trouxe incertezas ao futuro profissional desses alunos. Há riscos significativos de socialização profissional prejudicada devido à incongruência entre as expectativas dos alunos e a realidade dos departamentos clínicos
A5	2020 Annals of Medicine and Surgery	Dhahri et al.^17^	“O impacto psicológico do COVID-19 na educação médica de alunos do último ano no Paquistão: um estudo transversal”	Estudo transversal Amostra: 2661 estudantes de medicina e odontologia	Avaliar os efeitos psicológicos da pandemia de COVID-19 na educação médica do último ano médico e odontológico estudantes no Paquistão.	A pandemia por Covid-19 trouxe impacto psicológico signi_cativo nos estudantes de medicina e odontolo-gia, no entanto, apesar dos efeitos negativos, os mesmos mantêm o interesse em servir a comuni-dade.
A6	2020 Annals of Medicine and Surgery	Elsalem et al.^18^	Estresse e mudanças comportamentais com exames eletrônicos remotos durante a pandemia de Covid-19: um estudo transversal entre graduandos de ciências médicas	Estudo transversal Amostra: 1019 estudantes de Ciências Médicas na Jordânia	Avaliou a experiência de estudantes de E-exames remotos durante a pandemia de COVID-19 entre estudantes de Ciências Médicas na Jordânia	Observou impacto negativo na realização de exames eletrônicos. Método gerou estresse nos alunos, e conse-quentemente alterações em hábitos alimentares, atividade física e do sono principalmente.
A7	2020 Perspect Psychiatr Care	Ersin; Kartal.^19^	A determinação dos níveis de estresse percebidos e comportamentos de proteção à saúde de estudantes de enfermagem durante a pandemia COVID-19	Desenho descritivo Amostra: 372 alunos na enfermagem	Determinar os níveis de estresse percebi-dos e os comporta-mentos de proteção à saúde de estudantes de enfermagem durante a pandemia de COVID-19.	É comum que os níveis de stress aumentem pelo fato de ter que tomar medidas de proteção fora de casa, o covid-19 agrava o quadro
A8	2020 Nurse Education in Practice	Eweida et al.^20^	Tensão mental e mudanças no centro de saúde psicológica entre estudantes internos de enfermagem em unidades pediátricas e médico-cirúrgicas em meio à pandemia de COVID-19: uma pesquisa abrangente	Estudo transversal descritivo Amostra: 150 estudantes internos de enfermagem	Explorar o desgaste mental e as mudanças no pólo de saúde psicológica entre os estudantes internos de enfermagem durante a pandemia do COVID-19	A pandemia de COVID-19 foi identificada como uma grande fonte de tensão mental entre estudantes internos de enfermagem em unidades pediátricas e médico-cirúrgicas e teve um impacto negativo em sua saúde psicológica
A9	2020 International Journal of Environmen-tal Research and Public Health	Gallego-Gómez et al.^21^	A pandemia de COVID-19 e seu impacto sobre estudantes de enfermagem domiciliares	Estudo observa-cional e prospectivo Amostra: 138 estudantes de Enfermagem do segundo ano da Faculdade UCAM (Murcia, Espanha)	Avaliar os níveis de estresse de estudantes de enfermagem antes e durante o bloqueio devido à pandemia COVID-19 em Murcia (Espanha), sua influência na realização de um exame online e como foi afetado pelo exercício físico.	A quarentena gerou estresse pela expectativa do futuro da profissão. Alteração para ensino online somatizou o estresse, mas o desempenho acadêmico foi positivo. Desencadeou problemas financeiros, familiares e emocionais. Quem praticava atividade física teve menor nível de estresse.
A10	2020 Nutrients	Gallo et al.^22^	O impacto das medidas de isolamen-to devido ao COVID-19 na ingestão de energia e nos níveis de atividade física em estudantes universitários australianos	Estudo observacional Amostra: 509 estudantes de Biomedicina Universidade de Queensland (Brisbane, Austrália)	Avaliar o impacto das medidas de isolamen-to, incluindo a transição para o aprendizado online, nos padrões de dieta e atividade física.	Mudanças indese-jáveis na dieta e nos padrões de atividade física, especialmente se sustentadas por algum tempo, podem ter consequências deletérias para o bem-estar físico e mental.
A11	2020 Frontiers in Public Health	Khasawneh et al.^23^	Estudantes de medicina e COVID-19: Conhecimento, atitudes e medidas de precaução. Um estudo descritivo de Jordan	Estudo transversal descritivo Amostra: 1.404 estudantes de medicina	Avaliar o conhecimen-to, atitude, percepções e medidas de precaução em relação ao COVID-19 entre uma amostra de estudantes de medicina na Jordânia.	Alunos apresentaram boas medidas de preocupação e bom conhecimento sobre Covid-19. As informações obtidas obtiveram mais em mídias sociais em relação a fontes cientí_cas
A12	2020 Rev. Bras Promoç Saúde	Martins et al.^24^	Identificar a prevalên-cia do sentimento de angústia autorreferido e seus fatores relacionados, bem como a adesão ao isolamento social de universitários da área da saúde durante a pandemia da COVID-19	Estudo transversal Amostra: 541 universitários da área da saúde	Identificar a prevalên-cia do sentimento de angústia autorreferido e seus fatores relacionados, bem como a adesão ao isolamento social de universitários da área da saúde durante a pandemia da COVID-19	O Sentimento de angústia apresentado e esteve relacionado à preocupação com o mundo e serem favoráveis ao isolamento social,
A13	2020 Iranian Journal of Psychiatry	Nakhostin-An-sari et al.^25^	Depressão e ansiedade entre estudantes de medicina iranianos durante a pandemia de COVID-19	Estudo transversal Amostra: 323 estudantes de Universidade de Ciências Médicas de Teerã	Determinar os níveis de depressão e ansiedade entre estudantes de medicina iranianos durante a pandemia COVID-19.	Depressão e ansiedade não diferiram significativa-mente entre os estudantes de medicina iranianos antes e depois do surto COVID-19. Os sintomas somáticos da depressão são mais comuns durante esta pandemia
A14	2020 Journal of Medical Education and Curricular Development	Olum et al.^26^	Educação Médica e E-Learning durante a Pandemia de COVID-19: Conscien-tização, Atitudes, Preferências e Barreiras entre Estudantes de Graduação em Medicina e Enferma-gem na Universidade Makerere, Uganda	Estudo transversal online Amostra: 214 estudantes de Medicina e enfermagem em Uganda	Avaliar a consciência, atitudes, preferências e desafios para e-le-arning entre os alunos de Bacharelado em Medicina e Bacharelado em Cirurgia (MBChB) e Bacharel em Enferma-gem (B.NUR) na Makerere University, Uganda.	O uso de aulas e-learning é reconhe-cido pelos alunos, mas os resultados são negativos pelas barreiras que os alunos enfrentaram para essa modalidade de ensino.
A14	2020 BMC Medical Education	Puljak et al.^27^	Atitudes e preocu-pações de estudantes de graduação em ciências da saúde na Croácia em relação à mudança completa para o e-learning durante a pandemia COVID-19: uma pesquisa	Estudo observa-cional transversal Amostra: 2.520 alunos de ciências da saúde	Explorar as atitudes e preocupações dos estudantes de ciências da saúde na Croácia em relação à mudança completa para o e-learning durante a pandemia COVID-19.	A maioria dos estudantes referiram estar satisfeitos com o modelo de educação e-learning exclusivo durante a pandemia COVID-19
A15	2020 BMC Medical Education	Puljak et al.^27^	Atitudes e preocu-pações de estudantes de graduação em ciências da saúde na Croácia em relação à mudança completa para o e-learning durante a pandemia COVID-19: uma pesquisa	Estudo observacional transversal Amostra: 2.520 alunos de ciências da saúde	Explorar as atitudes e preocupações dos estudantes de ciências da saúde na Croácia em relação à mudança completa para o e-learning durante a pandemia COVID-19.	A maioria dos estudantes referiram estar satisfeitos com o modelo de educação e-learning exclusivo durante a pandemia COVID-19
A16	2020 International Journal of Environmen-tal Research and Public Health	Ramos-Morcillo et al.^28^	Experiências de estudantes de enfermagem durante a mudança abrupta da educação presencial para a educação on-line durante o primeiro mês de reclusão devido ao COVID-19 na Espanha	Abordagem qualitativa 32 Graduandos e mestrandos de enfermagem	Conhecer as experiências de aprendizagem e as expectativas sobre as mudanças na formação de estudantes de enfermagem matriculados nos cursos de bacharelado e mestrado em enfermagem de duas universi-dades públicas espanholas, diante da brusca mudança do presencial para o e -aprender a educação durante o primeiro mês de confinamento devido à pandemia COVID-19.	As mudanças na modalidade de ensino trouxeram limitações para alunos mais velhos, que possuem família e outras responsabilidades e ou que vivem em regiões rurais, devido às limitações nos recursos eletrônicos. Ensino online substitui teoria, mas não prática clínica.
A17	2020 RMHP	Saddik et al.^29^	Níveis aumentados de ansiedade entre estudantes universi-tários de medicina e não-médicos durante a pandemia de COVID-19 nos Emirados Árabes Unidos	Estudo transversal Amostra: 1.385 Alunos de medicina e odontologia da Universidade de Sharjah e alunos de outras universidades	Avaliar o sofrimento psicológico e as preocupações de estudantes universitários durante a recente pandemia de COVID-19, o grau de percepção das informações sobre a doença e a atitude, práticas e comporta-mentos gerais durante o surto.	A maioria dos alunos mostraram bom nível de conhecimentos vindos de fontes confiáveis. Os níveis de ansiedade foram variados entre leves a graves. Após transição para ensino online a ansiedade diminuiu.
A18	2020 Nurse Education in Practice	Savitsky et al.^30^	Ansiedade e estraté-gias de enfrentamen-to entre estudantes de enfermagem durante a pandemia covid-19	Estudo transversal Amostra: 244 alunos da Enfermagem	Avaliar os níveis de ansiedade e formas de enfrentamento entre estudantes de enfermagem no Ashkelon Academic College, Southern District, Israel.	O estudo demonstrou novas evidências sobre a ansiedade nos alunos. Os mesmos apresentaram ansiedade severa pelos efeitos da pandemia.
A19	2020 Perspect Psychiatr Care	Ugurlu et al.^31^	O exame da relação entre os níveis de depressão, ansiedade e estresse de estudantes de enfermagem e comportamentos alimentares restritivos, emocionais e externos no processo de isolamento social do COVID 19	Estudo de correlação Amostra: 411 estudantes de enfermagem	Examinar a relação entre ansiedade, depressão, níveis de estresse e comporta-mentos emocionais, externos e alimentares restritivos dos alunos no processo de doença coronavírus de 2019 (COVID 19).	No estudo, verificou-se que a alimentação emocion-al e os comportamen-tos alimentares externos aumentaram com o aumento da depressão dos estudantes de enfermagem, e a alimentação restritiva, a alimentação emocional e os comportamentos alimentares externos aumentaram com o aumento da ansiedade e do estresse dos alunos.
A20	2020 *Journal of Dental Education*	Umeizudike et al.^32^	Conhecimento, percepção e atitude de estudantes de odontologia nigeria-nos em relação ao COVID-19 e às práticas de controle de infecção	Estudo transversal Amo102 alunos de graduação em odontologia clínica	Avaliar o conhecimen-to, percepção e atitude dos alunos de graduação em odontologia na Nigéria em relação à pandemia COVID-19 e às práticas de controle de infecção.	Metade dos alunos tinha conhecimento adequado sobre Covi-19, mas apresentaram boa percepção e atitudes positivas em relação às práticas de controle da infecção.
A21	2020 BMJ Open	Wang et al.^33^	Associação entre experiências anteriores de estudantes de medicina e percepções da educação formal online desenvolvida em resposta ao COVID-19: um estudo transversal na China	Estudo transversal Amostra: 99 559 Alunos de medicina	(1) Compreender as características das experiências de aprendizagem online de estudantes chineses de graduação em medicina; (2) Investigar as percepções dos alunos sobre a educação online contínua desenvolvida em resposta ao COVID-19 e (3) Explorar como as experiências anteriores de apren-dizagem online estão associadas às percepções dos alunos.	Alunos da de medicina da China tem experiências em aulas online, o que resultou em satisfação. Mas no que se refere às fases de práticas clínicas, os escores de satisfação ficaram baixos.
A22	2020 Int J Environ Res Public Health	Xiao et al.^34^	Distanciamento social entre estudantes de medicina durante a pandemia da doença coronavírus de 2019 na China: conscien-tização sobre a doença, transtorno de ansiedade, depressão e atividades comportamentais	Estudo transversal Amostra: 933 estudantes de medicina	Compreender os efeitos psicológicos das medidas de distanciamento e os possíveis efeitos no bem-estar do estudante de medicina.	A pressão da saúde mental esteve acentuada no período da pandemia
A23	2020 Perspect Psychiatr Care	Yavas Çelik,^35^	O efeito de ficar em casa devido ao surto de COVID-19 na satisfação com a vida e nas competências sociais dos estudantes de enfermagem	Estudo transversal Amostra: 271 estudantes de enfermagem	Investigar a satisfação com a vida e as competências sociais de acadêmicos de enfermagem	Os alunos foram afetados pelo distanciamento, consideraram as medidas insuficientes para combate só covid-19 e a baixa satisfação com a vida

Quanto aos tipos de estudos encontrados, entre os selecionados, 74% (n=17) utilizaram o tipo observacional, sendo 88% (n=15) do tipo transversal e 6% (n=1) prospectivo. Ainda, 13% (n=3) realizaram estudo descritivo e 13% (n=3) apresentaram estudo de abordagem qualitativa.

Outra característica encontrada sobre os artigos selecionados foi o local de realização. Os estudos aconteceram em 15 países diferentes, sendo 95,7% internacional. A Turquia, Jordânia e Espanha se destacam com 13% dos locais de realização do estudo. O Brasil teve uma publicação representando o percentual de 4,3% dos estudos selecionados, e esse foi desenvolvido na cidade de Fortaleza no Ceará.

Para melhor compreensão dos desfechos encontrados nos artigos revisados, foi feito o agrupamento e classificação das variáveis, que resultou na apresentação de quatro categorias: (1) Efeitos relacionado a desordens nas competências emocionais, encontrado em 15 artigos; (2) ao ensino, presentes em 10 artigos; (3) à alimentação, sono e atividade física, em 8 artigos e (4) outras manifestações, descritos em 10 dos artigos da revisão. (Tabela 2)

**Tabela 2:** Distribuição de frequência de efeitos na rotina dos acadêmicos de ciências da saúde devido distanciamento social por covid-19

Variáveis	nº Expressos	% N=23	Autores
Efeitos relacionado a desordens nas competências emocionais			
Ansiedade moderada a grave	9	39.1%	A1, A3, A4, A8, A13, A17, A18, A19, A22
Depressão moderada a grave.	6	26%	A1, A5, A8, A13, A19, A22
Estresse	5	21,7%	A6, A7, A8, A9, A19
Sentimento de incerteza, medo do futuro e angústia	4	17.4%	A5, A12, A16, A18
Medos (de se infectar, transmitir para familiares,	3	13%	A3, A8, A18
desorganização do sistema de saúde, falta de Equipamento de Proteção Individual (EPI), enfrentar e gerenciar situações difíceis)			
Frustração, vontade de abandonar carreira, Sentimento de inutilidade, Nervosismo, assustados, raiva	1	4,3%	A8
Sentimento de isolamento	1	4,3%	A5
Efeitos relacionadas ao ensino			
Vantagens e satisfação com aulas e-learning exclusivo durante a pandemia COVID-19	4	17.4%	A2, A9, A15, A21,
Desvantagens: baixa qualidade do ensino, interação deficiente com os instrutores, limitações tecnológicas, incompatibilidade com prática, Universidade despreparada para ensino online	5	21,7%	A2, A5, A14, A16, A21
Carente ou preocupada com a falta de aulas práticas	1	4,3%	A15
Antecipação das atividades profissionais, visto como valorização e recompensa (antes de concluir o curso)	3	13%	A3, A4, A8
Efeitos relacionados à alimentação, sono e atividade física			
Problemas para dormir. Alterações do sono	5	21,7%	A5, A6, A7, A8, A13
Mudanças na rotina da atividade física, redução ou aumento das horas de atividade física	4	17.4%	A6, A7, A9, A10,
Mudanças hábitos alimentares	2	8.7%	A6, A19
Manifestações de natureza diversas, desenvolvidas no distanciamento social			
Problemas financeiros, familiares e emocionais	3	13%	A9, A12, A18
Falta de conhecimento cobre o Covid -19 e habilidades na prática profissional, Informações pelas mídias sociais	3	13%	A3, A11, A20
Pequena minoria de alunos, se infectados evitariam isolamento, Resistência ao uso de EPI	2	8.7%	A11, A20
Falta de prazer nas atividades diárias	1	4,3%	A5
Diminuíram (visitas sociais, aglomerações, aperto de mão, evitou pessoas com sintomas gripais) Uso de EPIs máscara, desinfetantes mãos, higiene das mãos)	1	4,3%	A17

Fonte primária: Informações extraídas dos estudos de revisão

Nessa tabela (Tabela 2), foi apresentado a síntese de como o distanciamento social em virtude da pandemia do vírus da Covid-19 afetou a rotina diária de estudantes de ciências da saúde. Também foi apresentado a frequência que cada variável teve entre os artigos.

## Discussão

Talvez o maior percentual de estudos presentes na literatura internacional seja justificado no fato de a ocorrência do primeiro caso de infecção por Coronavirus SARS CoV-2 ter sido na China,^11^ e em seguida sua rápida disseminação, agregou características de pandemia com Covid-9, onde os países mais afetados foram os asiáticos e europeus.^2^ Essas regiões vivenciaram inicialmente essa realidade estudada, o que pode justificar o grande volume de estudos encontrados sobre a temática.

Dos estudos analisados, 17,4% tiveram como público estudado, estudantes de ciências da saúde, e 82,6% para cursos específicos. O destaque ficou para os cursos de Enfermagem, Medicina e Odontologia. Supõe-se que o maior interesse por esses cursos esteja relacionado à preocupação com os alunos que se encontram na posição de futuros profissionais, que em situações de crise pandêmica, possuem maior exposição ao risco de contágio por provável atuação na linha de frente de combate.

O distanciamento social dos estudantes de ciências da saúde, segundo, 65,2% dos artigos analisados, acarretou consequências na competência emocional e, a manifestação da ansiedade esteve presente em 39,1% dos estudantes.

A ansiedade surge em situações de dificuldades de adaptação, já foi percebida em estudantes da área de ciências da saúde quando expostos a situações que por eles são identificadas como risco ou ameaça no ambiente universitário^36^, muito dos que apresentaram ansiedade, além das mudanças drásticas e necessárias nas modalidades de ensino, foi referida por ter amigos e conhecidos infectados por Covid-19.^13^

As incertezas no ambiente acadêmico, as medidas de segurança, bem como incerteza do futuro podem ser as causas que levaram estudantes a apresentar depressão, uns de forma moderada outros de forma mais grave.^13^^,^^17^^,^^20^^,^^25^^,^^31^^,^^34^

Estudo realizado com universitários em Portugal no início da Pandemia mostrou aumento significativo de ansiedade, depressão e estresse nesses estudantes em relação a anos anteriores, e já se alertava para o agravamento desses quadros, visto que a realidade pandêmica teria previsibilidade de agravamento.^37^ Nosso estudo mostra, que a soma das variáveis estresse, ansiedade e depressão, foram registrados em 87% dos estudos revisados. É importante observar a magnitude dessa informação, pois suas influências, favorecem para o desequilíbrio físico e mental, diminuindo assim o desempenho acadêmico.^38^

Na categoria que retrata as manifestações sobre os efeitos relacionados ao ensino, em 15,4% dos artigos houve o entendimento de satisfação sobre as mudanças das universidades para as aulas e-leaming, e que as mesmas trouxeram vantagens na interrelação com colegas, professores, facilidade em alguns acessos via tecnologias que não teriam se o ensino fosse presencial.^14^^,^^21^^,^^27^^,^^33^ Esse entendimento de que o ensino e-leaming foi vantajoso, foi manifestado por estudantes que tinham equipamentos e acesso à internet de boa qualidade e que não estavam necessitando cursar aulas práticas.

Em contraponto, 26% manifestaram desvantagem sobre a modalidade do ensino exclusivo online. Mudanças da modalidade de ensino presencial, para modalidade online de forma abrupta e sem planejamento prévio, provavelmente trouxe dificuldades, como financeiras ou tecnológicas e até geográficas, limitado os sinais de internet por exemplo. Também o ensino online não é compatível com aulas práticas. Essas circunstâncias aumentam situações de ansiedade, estresse, depressão, incertezas dentre outras.^7^

A necessidade de mão de obra para o enfrentamento da pandemia, serviu de argumento para algumas universidades abreviarem o tempo de alguns cursos da área da saúde, antecipando a inserção desses acadêmicos no mercado de trabalho. Esse evento, foi entendido por muitos alunos como uma vantagem ou oportunidade ou ainda valorização pelo papel que exercerá na situação de crise pandêmica.^15^^,^^16^^,^^20^

Outra manifestação evidenciada entre os estudantes, refere-se aos problemas relacionados com o sono. Nesse estudo, o distúrbio do sono teve sua frequência apontada por 19,2%, entretanto, ressalta-se que vários estudos dessa revisão, apontaram para inúmeras circunstâncias que estão associadas ao comportamento do sono, como por exemplo, estresse, depressão, preocupações financeiras, medos, distanciamento social, incertezas, mudanças na modalidade de ensino dentre outros. Essas manifestações também são descritas por Blume; Schmidt; Cajochen, Sher,^39^^,^^40^ como fatores associados aos distúrbios do sono.

O distanciamento social também trouxe influências na rotina da atividade física dos estudantes. Em 17,4% dos estudos, observou-se a manifestação de mudanças na sua atividade física, sendo que 75% foi de redução em relação ao período anterior às medidas restritivas. Também houve mudanças nos hábitos alimentares em 8,7% dos estudos. A atividade física moderada a intensa possui forte associação com a qualidade do sono, redução do estresse e bem-estar geral. Assim também, uma alimentação balanceada fortalece o organismo a se manter em equilíbrio, físico, psíquico e imunológico, agregando melhores condições para vida em isolamento social.^41^^,^^42^

Outras manifestações de natureza diversas também foram identificadas. Mas destaca-se os problemas de ordem financeira, e familiares. O somatório do ensino remoto, filhos para cuidar, emprego ameaçado e riscos de serem infectados, desencadeou instabilidade emocionais, sinalizando à necessidade de intervenção, para auxiliar os alunos na superação desse momento de crise pandêmica.^21^^,^^24^^,^^30^

Para alguns estudantes, a preferência pela busca do conhecimento e informações sobre a Covid-19 foi através das mídias sociais. Os resultados dessa opção de busca ao invés de meios científicos, pode ser a causa de que 13% dos estudantes apresentaram conhecimento insatisfatório sobre a doença. Associado a isso, pode-se inferir também a falta de habilidades e despreparo para atuação profissional, principalmente pelo fato de ainda não ter concluído efetivamente seu curso.^15^^,^^23^^,^^32^

Apesar da pandemia por Covid-19 ter demandado mão de obra além do esperado, a antecipação da inserção na vida profissional de estudantes da área da saúde merece atenção especial. Há necessidade de ser observado a real habilidade e conhecimento sobre a clínica desses alunos e de onde suas fontes de informações são extraídas. Para atuar com seres humanos, mesmo em momentos de crise, é necessário possuir bases sólidas, estruturadas em evidências e na ciência.^15^

Vale destacar, que 4,2% de uma amostra de estudantes de ciências da saúde admitiu que se caso fosse infectado pelo SARS-CoV-2, faria de tudo para evitar o isolamento social, e também em 8,7% dos estudos, houve demonstração de aluno com resistência ao uso de EPI como estratégia de prevenção à infecção do vírus da Covid-19.^23^^,^^32^ São comportamentos, que apesar de representar a minoria dos alunos participantes dos estudos analisados, não devem ser desconsiderados, principalmente por se tratarem de estudantes de ciências da saúde e futuros profissionais, formadores de opiniões.

## Conclusão

Essa revisão demonstrou que o distanciamento social influenciou negativamente na rotina dos estudantes de ciências da saúde. A abrangência das influências foi relacionada a múltiplos fatores. Mas se destacam as que provocaram desordens nas competências emocionais, como a ansiedade, depressão, estresse e incertezas sobre o futuro e sobre as consequências da pandemia.

Conhecer como o isolamento social influenciou na rotina dos estudantes de ciências da saúde é relevante para que as universidades desenvolvam programas de suporte para as necessidades apresentadas na realidade atual. Também refletir sobre as consequências da pandemia atual servirá para projetar, nas instituições de ensino superior, programas de enfrentamento para futuras crises epidêmicas, e que auxiliem a minimizar os impactos resultantes da mesma.
